# RNA editing‐based biomarker blood test for the diagnosis of bipolar disorder: results of the EDIT-B consortium study

**DOI:** 10.1192/j.eurpsy.2026.10189

**Published:** 2026-07-31

**Authors:** D. Weissmann, N. Salvetat, F. J. C. Robles, C. Cayzac, F. S. Schneider, M. Menhem, D. Vetter, J. M. Haro, C. Henry, L. V. Kessing, E. Vieta

**Affiliations:** 1 Parc Euromédecine, ALCEDIAG; 2 UMR 9005 CNRS/ALCEN, Parc Euromédecine, Sys2Diag, Montpellier, France; 3 Sant Joan de Déu, Parc Sanitari, Barcelona, Spain; 4 Psychiatrie & Neurosciences, GHU Paris, Paris, France; 5 Psykiatrisk Center, København, Denmark; 6 Hospital Clinic de Barcelona, Barcelona, Spain

## Abstract

**Introduction:**

Differentiating bipolar disorder (BD) from major depressive disorder (MDD) remains a major challenge in psychiatry. Although both present with depressive episodes, their underlying mechanisms and treatment pathways differ significantly. As BD often begins with depression, diagnostic approaches relying on symptoms and interviews lack precision, leading to the misdiagnosis of BD patients as MDD. The average diagnostic delay (about 10 years) has serious clinical consequences, including inappropriate treatment, risk of manic switching and increased suicide rates. A-to-I RNA editing is a highly dynamic process implicated in regulatory mechanisms relevant to psychiatry. Recent studies used circulating RNA editing biomarkers to generate AI-based algorithms capable to differentiate BD from MDD patients (Salvetat et al., 2024, J. Affect. Disord. 356, 385–393).

**Objectives:**

This study aimed to externally validate and compare the diagnostic performance of three previously developed algorithms for distinguishing BD from MDD in patients experiencing a major depressive episode in a multicentric cohort.

**Methods:**

In this multicenter cross-sectional trial (NCT05603819), adults with BD or MDD presenting a current depressive episode (N=393) were recruited across four sites in Barcelona, Paris, and Copenhagen. Blood samples were collected, RNA was extracted, and sequencing data were processed via a secure HDS-certified platform. Algorithm outputs were compared with physician diagnoses (MINI, MADRS, YMRS and EQ-5D-5L).

**Results:**

The three algorithms were evaluated and compared using ROC analysis. Observed AUC were 0.834 (CI 95%: [0.793 - 0.875]) for Algorithm A1, 0.873 (CI 95%: [0.837 - 0.909]) for Algorithm A2, and 0.741 (CI 95%: [0.690 - 0.792]) for Algorithm A3. Algorithm A2, exhibited the strongest results and highest performance, with high sensitivity (82.6%) and specificity (80.3%). These results confirm our previous findings and reproduce the performances obtained on a previous independent cohort (Salvetat et al., 2024, J. Affect. Disord. 356, 385–393).

**Conclusions:**

The study confirms the diagnostic potential of RNA editing signatures combined with AI for differentiating BD from MDD. Algorithm A2 demonstrated robust and reproducible accuracy in three independent cohorts, corroborating its value as an innovative complementary diagnostic tool in clinical practice.

**Disclosure of Interest:**

D. Weissmann Grant / Research support from: Part of the funding for this study was provided by EIT Health., N. Salvetat: None Declared, F. ROBLES: None Declared, C. Cayzac: None Declared, F. SCHNEIDER: None Declared, M. MENHEM: None Declared, D. Vetter: None Declared, J. Haro: None Declared, C. Henry: None Declared, L. Kessing: None Declared, E. VIETA: None Declared

